# What Kills the Children

**Published:** 1889-05

**Authors:** 


### SELECTIONS.

### What Kills the Children.

Ignorance kills the children. Now and then accident lends a hand, as when some poor toddler tumbles into boiling water, or a weary, restless mother rolls upon her infant and stills its cries forever. But ignorance does the business generally, blind giant that he is, always ready to seize the pillars of every thing and turn bright places of joy as dark as buried Babylon. Tradition clutches the hapless infant the moment it is born, and decrees that it shall be immersed in water. The prenatal environment has a temperature of about a hundred degrees. This cold world boasts of any temperature it pleases. That of the bath is also a movable feast. Under the circumstances, the custom of washing the new-born is not unlike, in effect, the refinement of cruelty that would grasp a man from his bed and thrust him ruthlessly into a snow-bank. The fact that childbirth is an exhausting process, even to the baby, is too often ignored. Time is required for necessary reaction. The little one needs repose as well as its mother, a good rest at the start to insure some health and strength for its future guerrilla warfare with life. If it were let alone, sleep would generally follow its preliminary outcries. But no; custom says the little pilgrim must be scrubbed, and scrubbed he is. He survives—as a general thing. But "How did my lamb get that dreadful cold and those bad eyes?" and "How he cries with colic!" It is possible to manage better, as for instance, in the Maternity Department of the Woman's Hospital in Philadelphia. There the eyes of new-born babies are washed with an antiseptic solution at the earliest opportunity. When the child is entirely born, it is left quiet on the mother's bed until the expulsion of all appendages, which usually occurs within an hour. The infant is then carried away just as it is, well wrapped up, and let alone. After the mother has received proper attention the child is cared for. One loose garment and a diaper are all it is burdened with in the way of clothes. Warmly covered, it is laid away in a little bed by itself, and neither washed nor dressed until twenty-four hours have elapsed, when it is carried to the baby's bath-room (which is properly heated), and there its toilet is performed for the first time. The physician in charge states that since this plan has been adopted the babies thrive to a far greater degree and cry less. This method is at once rational and natural, and would seem to need no defense

The maternal physique has some subtle, indefinable influence over young children, a health-giving power not at present well understood. The new baby is still in a certain sense a part of its mother, although a separate unit. Its well-being requires close contact with her during the greater part of the twenty-four hours. A bed by itself is an injustice to helpless infancy. It is paterfamilas who should seek another resting place, not the new life as yet so frail and insecure. Only those who have tried this natural method can thoroughly appreciate its advantages and realize how admirably it insures the happiness of three persons. The child can be cared for during the night without exposure or any sudden chill. Always warmed and protected by a loving presence, the little one sleeps long and well. After the weaning period, the baby has his own bed as a matter of course. Until then, an undisputed half of the maternal couch is a necessity to the embrionic citizen, if he is to grow into that relative perfection of health and strength which nature has intended for him. The human mother is the only animal that puts away its young at night, probably because the right kind of reason has not yet taken the place of half-eradicated instinct. The hen gathers her brood under her wings; the mother bear forms herself into a sort of animate woolly nest ahout her cubs, just as the cat's body embraces her kittens. Our cousins of the lower orders may not be such bad examples to follow after all. At any rate, why not give those "wonderfu' weans" the benefit of the doubt?

The slaughter of the innocents goes on in different ways. Emotional prodigality is a most efficient means of removing the joys of a household. "Died of too much grandfather, grandmother, uncle, and aunt" would be a fitting epitaph for many a bright child. Emotion is the most exhausting of all mental attributes. What children do, and how much, is of far less importance than the way in which they do it. The evils of premature mental activity are without doubt very great; but to prematurely and unduly excite emotional manifestations is tenfold more hurtful. In this regard there seems to be the densest ignorance. The fact that young children's only business in life is to develop slowly—to eat, sleep, and play in childlike fashion —is too often forgotten in the home circle. On the contrary, they are supposed to attend to their own work of growing and developing, and afford fun for the family at the same time. Our tender little ones are made the play-things of the household—hugged, kissed, talked to, and made to talk, for the pleasure and gratification of the parents and friends. Their callow brains are overworked by exciting and intense emotion. What wonder they have big heads, little bodies, and hardly any digestion at all! Feebleness, asymmetry, excitability, premature arrest of growth, are some of the evils resulting from this continual tension selfishly imposed by thoughtless grown folk upon unresisting childhood. To what extent the influences under consideration can reach is, perhaps, known only to those who are in actual contact with large numbers of children, and who have made the subject of very young Americans something of a study. * * * *
—*The New York Medical Journal.*

